# Refractive Errors of School Children from Economically Disadvantaged Areas in Northwest México

**DOI:** 10.3390/jcm13113094

**Published:** 2024-05-25

**Authors:** Emiliano Teran, Efrain Romo-García, Hector C. Santiago

**Affiliations:** 1 Faculty of Physical-Mathematical Sciences, Autonomous University of Sinaloa, Culiacan 80246, Sinaloa, Mexico; 2 Department of Ophthalmology, Center for Research and Teaching in Health Sciences, Autonomous University of Sinaloa, Culiacan 80246, Sinaloa, Mexico; efrainromo@hotmail.com; 3 School of Optometry, Inter American University of Puerto Rico, 500 John Will Harris, Bayamón, PR 00957, USA; hsantiago@opto.inter.edu

**Keywords:** refractive errors, myopia, hyperopia, astigmatism, vision screening, pediatric ophthalmology

## Abstract

**Background**: Refractive errors, including myopia, hyperopia, and astigmatism, are the leading causes of visual impairment in school-aged children and can significantly impact their academic performance and quality of life. This study aimed to assess the prevalence of refractive errors among school children from economically disadvantaged areas in Northwest México, using a consistent methodology to facilitate comparison with global data. **Methods**: We adopted the Refractive Error Study in Children (RESC) protocol by the World Health Organization to examine the prevalence of myopia, hyperopia, and astigmatism. The study comprised a systematic sampling of children aged 6 to 18 years from diverse schools in Northwest México. Trained optometrists conducted visual acuity testing and autorefraction, while ophthalmologists performed cycloplegic refraction to ensure accuracy. **Results**: The study found a myopia (SE ≤−1.50 D at least one eye) prevalence of 14.55% (95% CI: 13.27–15.91), with a higher incidence in females (6.92%) compared to males (6.00%) in at least one eye. Hyperopia (SE ≥ +1.00 D at least one eye) was less common, at 3.23% (95% CI: 2.61–3.95), with a slightly higher occurrence in males in at least one eye. Astigmatism (Cylinder ≥ 0.75 D at least one eye) was present in 18.63% (95% CI: 17.21–20.12) of the students in at least one eye, with no significant difference between genders. These findings are consistent with other studies in regions such as Puerto Rico and Iran, indicating widespread refractive error issues among schoolchildren. **Conclusions**: The high prevalence of refractive errors, particularly myopia and astigmatism, highlights the critical need for regular vision screenings in schools and the implementation of public health interventions to provide corrective eyewear. Our study confirms the importance of utilizing standardized methodologies like the RESC protocol to compare refractive error prevalence across different geographical and socio-economic contexts, thereby informing global public health strategies.

## 1. Introduction

Refractive errors, including visual conditions such as myopia, hyperopia, and astigmatism, significantly affect a child’s academic performance and overall well-being [1]. In socioeconomically marginalized areas, notably specific regions in Mexico, the prevalence of these visual anomalies is strikingly high, akin to other regions where refractive error rates reach up to 58% [2,3]. The consequences of unaddressed refractive errors in these areas are extensive. Evidence suggests that children with untreated visual impairments consistently underperform in academics, experience social isolation, and even reduce participation in physical activities, emphasizing the urgency for early detection and intervention [4].

While substantial efforts have been made globally in addressing refractive error correction, significant gaps remain. In Mexico, even with initiatives like “Ver bien para aprender mejor” distributing spectacles to numerous children annually [5], it was observed that only 74.5% of the myopic children wore their spectacles during examinations. Yet, comprehensive data mapping the refractive status of these children is scarce [6]. This gap in the existing literature emphasizes the need to deeply investigate the prevalence of these errors.

The northern region of Mexico stands out as one of the areas most affected by visual impairments. For instance, studies from Nuevo León reveal a striking prevalence of myopia at 46%, with hyperopia and astigmatism following closely. Similarly, in Sinaloa, the situation is alarming with myopia rates at 36%, hyperopia at 5%, and astigmatism at 22%. Considering the need to assess and compare the prevalence of refractive errors both regionally and globally, it becomes imperative to adopt a standardized and consistent methodology across studies. The Refractive Error Study in Children (RESC) offers an ideal solution to this challenge [7].

The RESC protocol is a well-established clinical standard that offers a comprehensive framework to examine the prevalence of refractive errors and the broader visual health in children. Its robustness and reliability have enabled it to be used as a primary tool to compare the prevalence of myopia across varied populations and nations. Examples of its widespread application include countries like Iran [8], Ghana [9], Chile [10], Colombia [11], Ireland [12], Saudi Arabia [13], and Tunisia [14]. Yet, a significant data gap remains regarding the overarching prevalence of refractive errors across Latin America, which is vital for the formulation of strategies aiming at enhancing visual health.

Addressing these challenges, this epidemiological study sets forth one primary objective: to quantify the prevalence of refractive errors among children in socio-economically disadvantaged areas. This study assessed students’ refractive status and spectacles compliance in the primary educational levels of Sinaloa, Mexico. Our project represents the inaugural epidemiological investigation into students’ visual impairments within a specific age range in Mexico, employing an internationally recognized protocol sanctioned by the World Health Organization.

## 2. Materials and Methods

We evaluated a total of 2422 subjects. Among these, 1241 (51.24%) were females, and 1181 (48.76%) were males. The children we assessed were aged between 6 and 12 years. Our study was conducted from July 2022 to June 2023 in Culiacan, Sinaloa, Mexico. Rather than adopting a random sampling method, we strategically selected schools based on their geographical locations as informed by city hall evaluations and the willingness of school authorities to cooperate. Our approach was grounded in the RESC protocol. Before commencing the study, we provided all participants with an informed consent letter. Before including children in our research, we secured permission from each child’s parent or guardian. Additionally, we ensured that the children themselves provided their assent, respecting their agency in the study. The Autonomous University of Sinaloa’s institutional review board granted approval for our study. Furthermore, our research rigorously followed the ethical principles set forth in the Declaration of Helsinki.

At the school premises, optometrists first assessed the children’s refractive status and visual acuity using the HOTV logMAR chart, placed at a 3 m distance. Children who demonstrated a distant visual acuity worse than 20/40 in either eye were subsequently referred for a comprehensive evaluation at the Vision Hospital, Hospital Buena Vista, a specialized eye care center. At this facility, ophthalmologists conducted detailed examinations, including cycloplegic refraction following the Refractive Error Study in Children protocol. We procured both wet and dry objective refraction measurements using an autorefractometer.

We placed paramount importance on the protection and confidentiality of the children’s data. Each participant was assigned a unique code, ensuring anonymity throughout the study. Their names were never used in the research documents or analyses. All personal information was securely stored in encrypted files to maintain privacy and adhere to ethical standards regarding children’s data protection.

We utilized the R statistics packages (version 1.3.1.1093; R statistics, Auckland, New Zealand [15]) to conduct mixed-effects regression analyses, with the spherical equivalent serving as the dependent variable. Visual acuity and demographic information were treated as independent variables, while the eye was considered a random variable. For data with nonparametric distributions, the Wilcoxon rank sum test with continuity correction was applied. We considered values with p≤0.05 to be statistically significant.

## 3. Results

**Figure 1:**
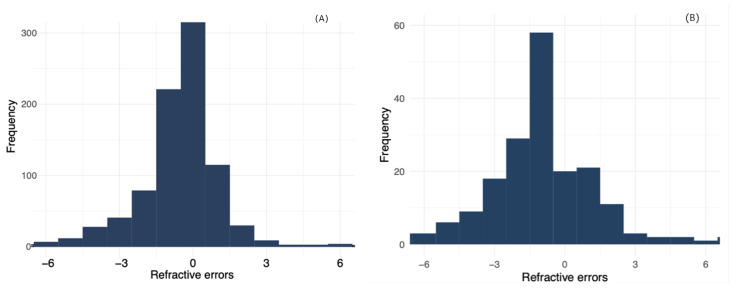
Uncorrected (**A**) and corrected (**B**) refractive errors of children students.

Figure 1 shows the refractive status of the subjects. Figure 1A shows the proportion of the distribution of the refractive errors found in subjects wearing no habitual correction. Likewise, Figure 1B shows the distribution of the refractive errors found in subjects who currently wear spectacles.

The Figure 1A illustrates the distribution of refractive errors, measured in diopters, across the children population. The x-axis spans from −1 to +6 diopters, encompassing ranges of myopia and hyperopia. The y-axis represents frequency per number of eyes. The distribution is left-skewed, with the majority of observations clustered around mild myopia, peaking between −1 and −1 diopters. The occurrences of moderate to high myopia, as well as hyperopia, are comparatively lower, as evidenced by the decreasing frequency of these conditions at the tails of the distribution. This suggests that mild myopia is the most prevalent refractive error within the sample.

Figure 1B presents the distribution of refractive errors in a sample of spectacles wearers, charted with the spherical equivalent in diopters on the x-axis against frequency on the y-axis. The diopter range spans from −1 to +6, encompassing both myopic and hyperopic corrections. The histogram peaks at −1.50 D, suggesting a predominance of mild myopia within this cohort. The data reveals a notable aggregation around the −1.50 D mark, indicating that mild myopia is the most common refractive error among the participants, rather than a balanced distribution of myopic and hyperopic corrections. The decreased frequency of higher magnitude refractive errors conforms with established patterns where moderate and high refractive errors are less prevalent. This histogram indicates a skewed distribution, with myopia being more frequent than hyperopia in this particular population of spectacles wearers.

Statistics pertaining to the prevalence of myopia are detailed in Table 1. This condition is now seen to affect 14.55% of the population, with a prevalence that is slightly more common in females (6.92%) than in males (6.00%). This gender disparity underscores a subtle yet significant difference in the occurrence of myopia between males and females. Bilateral myopia, where both eyes are nearsighted, occurs in 6.21% of the population, with a higher incidence in females (3.09%) compared to males (2.52%). Clinically significant myopia, which requires corrective measures for daily functioning, is present in 11.00% of individuals, with females (5.32%) again showing a higher prevalence than males (4.68%). High myopia, which is the extreme end of the condition, is comparatively uncommon, affecting 1.24% of the population, with females (0.85%) experiencing it more than five times as frequently as males (0.35%).

Hyperopia is significantly less common than myopia, affecting 3.23% of the population. The distribution between genders shows a reversal from myopia, with males (1.67%) having a higher prevalence than females (1.38%). This suggests that while myopia is more common, especially among women, hyperopia tends to be more prevalent in men, albeit to a lesser extent.

Astigmatism is present in 18.63% of the population, making it more common than myopia but less so than emmetropia. The gender distribution for astigmatism is relatively balanced, with males (9.05%) and females (8.30%) having similar prevalence rates. This indicates that astigmatism affects both genders nearly equally $\left(\chi^{2}=2.008,p=0.1565\right)$, likewise myopia ($\chi^{2}=0.1308,p=0.71)$ and hyperopia ($\chi^{2}=2.8228,p=0.09$), which do not show significant gender discrepancies.

Emmetropia, the condition of having normal vision without refractive error, is observed in 78.11% of the population. This condition represents the majority, with a slight female majority (37.62%) compared to males (38.82%).

## 4. Discussion

In this study, we evaluated the refractive errors among students aged 6 to 12 years in disadvantaged schools in Sinaloa, Mexico, utilizing a modified protocol based on the Refractive Errors Study in Children developed by the World Health Organization. The aim was to ascertain the prevalence of refractive errors within this demographic as per the guidelines of the World Health Organization.

The investigation revealed a myopia prevalence of 14.55%, higher in females (6.92%) than males (6.00%). Bilateral myopia was present in 6.21%, clinically significant myopia in 11.00%, and high myopia in 1.24%, with females more affected in each category. Hyperopia occurred in 3.23% of students, slightly more in men, and astigmatism in 18.63%, with a balanced gender distribution. Emmetropia was prevalent in 78.11%, with females (38.82%) slightly more than men. These results highlight gender-specific refractive needs, with a particular focus on myopia.

The prevalence of myopia among schoolchildren, particularly within economically disadvantaged regions, has emerged as a significant public health concern. Our study indicates that myopia occurs in 14.55% of children, with a higher incidence among females (6.92%) than males (6.00%). These findings are in line with the work of Teran et al. in Ref. [16], who reported a high prevalence of myopia at 36.11% in adolescents of the same region, suggesting the necessity of optometric examinations for students before university entry. The prevalence of bilateral myopia at 6.21%, clinically significant myopia at 11.00%, and high myopia at 1.24% in our study further echoes the global trend of increasing myopia prevalence [16]. Moreover, our data are consistent with findings from other regions of Mexico [17] and worldwide, including Puerto Rico [18], where a myopic shift with age was noted, with females showing a higher prevalence than males. Interestingly, Santiago et al. in Ref. [18] reported a 20.7% prevalence of myopia in Puerto Rican children, which is among the highest globally, emphasizing the need for monitoring and potential treatment for myopic progression. These observations underscore the gender disparity in refractive errors, which is crucial for public health initiatives.

Comparatively, studies in different geographical locations such as Ghana [9], Iran [8,19], and other parts of the Middle East [20] report varied prevalence rates. For example, myopia prevalence among Ghanaian schoolchildren was recorded at a low 3.2%, compared to 4.3% in Northeastern Iran, underscoring regional disparities as assessed through the RESC protocol. These discrepancies may reflect differences in genetic predisposition, environmental factors, and socioeconomic status, influencing the need for tailored public health policies for vision care.

The high prevalence of myopia and other refractive errors in schoolchildren from disadvantaged areas in Northwest Mexico, as well as globally, indicates an urgent need for regular eye examinations, accessible vision correction services, and further epidemiological research to understand the underlying causes and to develop effective interventions [8,9,16,18,19,20,21].

The prevalence of emmetropia underscores the fact that while refractive errors are common, a significant majority of the population possesses or achieves normal vision, either naturally or through correction.

In our study, a single visual acuity chart was utilized for all participants across different age groups to maintain consistency in measurement instruments. However, it is essential to acknowledge this approach as a potential limitation, especially given the variations in visual acuity development with age. Birch et al. [22] have highlighted the importance of selecting appropriate visual acuity protocols for specific populations. In their long-term follow-up of amblyopic children, two distinct visual acuity protocols, the Amblyopia Treatment Study HOTV (ATS HOTV) and the Electronic-Early Treatment of Diabetic Retinopathy Study (E-ETDRS), revealed slight discrepancies in visual acuity estimates, particularly in poorer acuity levels. The ATS HOTV protocol tended to slightly overestimate visual acuity compared to the E-ETDRS protocol, underscoring the potential for variations in assessment outcomes based on the chosen protocol. This insight underscores the necessity for careful consideration and potentially integrating multiple assessment tools, especially when evaluating populations with a broad age range, to ensure accurate and reliable visual acuity measurements.

Our study found a high astigmatism prevalence of 18.63% among schoolchildren in Northwest México, matching the 19.8 reported in Sinaloa’s younger students [21] and slightly less than the 29.17% in older adolescents [16]. This prevalence points to astigmatism as a significant refractive issue in Mexican children, similar to global trends. The uniformity across genders suggests that vision care initiatives should be inclusive of all children. High myopia rates elsewhere in the Americas, such as 20.7% in Puerto Rico [18], underscore the broader relevance of these findings. The data calls for integrated school vision screenings to correct refractive errors early, aiming to support children’s educational prospects and overall well-being. These results advocate for equitable access to eye care, ensuring that visual health is not a barrier to education for children in under-served communities.

## 5. Final Remarks

In the study of refractive errors among schoolchildren from economically disadvantaged areas in Northwest México, the prevalence of myopia was found to be 14.55%, and is particularly higher in females. This aligns with global observations of myopia as a common refractive error among the youth, necessitating early vision screening and correction to prevent educational impediments. Hyperopia, though less prevalent at 3.23%, showed a slightly higher occurrence in males, and astigmatism was observed in 18.63% of the children, distributed equally between genders. These findings underscore the critical need for regular optometric evaluations to address these refractive errors promptly.

The uniform application of the Refractive Error Study in Children (RESC) protocol, as utilized in this study, is crucial for ensuring comparability of results across different regions and studies. It allows for standardized data collection and analysis, facilitating the identification of patterns and trends in refractive errors on a global scale. Such methodological consistency is paramount for informing public health policies and designing targeted interventions that can effectively address the visual health needs of school-aged children, ultimately supporting their right to education and well-being.

## Figures and Tables

**Table 1:** The mean and 95% CI values for myopia, bilateral myopia, clinically significant myopia, high myopia, hyperopia, astigmatism, and emmetropia were analyzed for 2422 children attending clinics in Sinaloa, Mexico.

Group	*Definition*	Total	Male	Female
Myopia	SE ≤ −1.50 D at least one eye	14.55% (95% CI: 13.27–15.91)	6.00% (95% CI: 5.15–6.94)	6.92% (95% CI: 6.01–7.92)
Bilateral Myopia	SE ≤ −1.50 D in both eyes	6.21% (95% CI: 5.35–7.17)	2.52% (95% CI: 1.97–3.17)	3.09% (95% CI: 2.48–3.79)
Clinically Significant Myopia	SE ≤ −1.75 D at least one eye	11.00% (95% CI: 9.87–12.21)	4.68% (95% CI: 3.93–5.53)	5.32% (95% CI: 4.52–6.22)
High Myopia	SE ≤ +5.00 D at least one eye	1.24% (95% CI: 0.87–1.72)	0.35% (95% CI: 0.17–0.65)	0.85% (95% CI: 0.55–1.26)
Hyperopia	SE ≥+1.00 D at least one eye	3.23% (95% CI: 2.61–3.95)	1.67% (95% CI: 1.23–2.21)	1.38% (95% CI: 0.99–1.89)
Astigmatism	Cylinder ≥ 0.75 D at least one eye	18.63% (95% CI: 17.21–20.12)	9.05% (95% CI: 8.01–10.17)	8.30% (95% CI: 7.31–9.38)
Emmetropia	−1.50 < SE < +0.50 D	78.11% (95% CI: 76.53–79.62)	37.62% (95% CI: 35.82–39.43)	38.82% (95% CI: 37.02–40.65)

## Data Availability

Data are contained within the article.
